# Five traits of personality in benzodiazepine-dependent patients with bipolar disorder

**DOI:** 10.1192/j.eurpsy.2021.542

**Published:** 2021-08-13

**Authors:** N. Charfi, M. Ben Abdallah, S. Omri, R. Feki, N. Smaoui, L. Zouari, J. Ben Thabet, M. Maalej Bouali, M. Maalej

**Affiliations:** 1 Psychiatry C Department, Hedi chaker university hospital, sfax, Tunisia; 2 Department Of Psychiatry (c), CHU Hedi CHaker hospital Sfax Tunisia, Sfax, Tunisia

**Keywords:** traits of personality, benzodiazepine, dependence, bipolar disorder

## Abstract

**Introduction:**

Benzodiazepines (BZD) are frequently prescribed to patients with bipolar disorder. The use of this medication can become problematic in some cases or even lead to dependence mainly in patients with personality disorders.

**Objectives:**

To assess different personality traits in BZD-dependent patients with bipolar disorder

**Methods:**

A cross-sectional descriptive and analytical study was conducted on euthymic bipolar patients (DSM-5) attending the psychiatric outpatient in Hedi Chaker university hospital of Sfax. We used the Benzodiazepine Cognitive Attachment Scale (ECAB) to determine dependent patients and the Ten Item Personality Measure (TIPI) scale to assess the five personality traits.

**Results:**

Among the 61 included patients, 34 were BZD-dependents (55.7%). Their mean age was 52.12 ± 12.87 years. The sex ratio (M/F) was 0.54. A comorbid personality disorder was found in 18% of patients and the most frequent was histrionic personality (45.45%). According to TIPI, the highest scores of personality traits were conscientiousness, agreeableness and extraversion, with mean scores of 9.8 ± 2.78, 8.21 ± 3.15 and 7.98 ± 1.77 respectively. Having a comorbid personality disorder was not associated with BZD dependence. Regarding the dimensional approach using TIPI, the dimensions of extraversion, agreeableness and emotional stability were significantly negatively correlated with BZD-dependence (p 0.024; 0.006 and 0.01 respectively).

**Conclusions:**

This study demonstrates that personality characterized by less extraversion, agreeableness, and emotional stability may increase the risk of BZD-dependence among bipolar patients. Such psychological factors should be taken into account in the risk-benefit assessment made in the planning of BZD treatment.

